# The Effect of Biocontamination on Mechanical Strength and Moisture Transfer Performance of Epoxy Basalt Fiber Reinforcement Bar Exposed to Arctic Conditions

**DOI:** 10.3390/polym17040460

**Published:** 2025-02-10

**Authors:** Anatoly K. Kychkin, Oleg V. Startsev, Mikhail P. Lebedev, Aisen A. Kychkin, Irina G. Lukachevskaia

**Affiliations:** 1 Siberian Branch of the Russian Academy of Sciences V.P. Larionov Institute of Physical and Technical Problems of the North, 1 Oktyabrskaya Street, Yakutsk 677980, Russia; kychkinplasma@mail.ru (A.K.K.); startsevov@gmail.com (O.V.S.); 2 Siberian Branch of the Russian Academy of Sciences Federal Research Center «Yakut Scientific Center SB RAS», 2 Petrovskogo Str., Yakutsk 677000, Russia; m.p.lebedev@mail.ru (M.P.L.); icen.kychkin@mail.ru (A.A.K.)

**Keywords:** basalt-fiber-reinforced polymer (BFRP), Arctic exposure, biological contamination, strength, modulus of elasticity, moisture diffusion coefficient

## Abstract

This study involved the exposure of epoxy-coated basalt-plastic rebars, with diameters of 6 and 8 mm, to the open climate conditions of Yakutsk and Tiksi, located in the Arctic region of Russia. The exposure duration was 54 months. Basalt-plastic rebars were tested both untreated and after contamination with a set of neutral microorganisms resilient to cold climates, including spore-forming bacteria from the genus *Bacillus*, and mold fungi from the genera *Aspergillus*. Results showed that after 12, 24, and 54 months of exposure, the tensile strength and modulus of elasticity of untreated rebars increased by 5–14% due to the post-curing of the epoxy matrix. However, in biologically contaminated rebars, these indicators decreased on average by 11%. Bacterial cells and fungal mycelium, which penetrated surface irregularities of the rebars under open climate conditions, contributed to microcrack development, reducing the mechanical properties of the basalt-plastic rebars and causing additional moisture diffusion in the radial direction of the bars.

## 1. Introduction

Basalt-fiber-reinforced polymers (BFRPs) belong to the class of polymer composite materials widely used in engineering and construction [1,2,3]. Structures made from these materials are designed for long-term use in open climatic conditions [4,5,6], including in Arctic regions [7]. The review in Ref. [8] provides a detailed analysis of the chemical reactions and physical transformations in BFRPs under the influence of temperature, humidity, thermal cycles, chemically active environments, and other external factors. Processes such as plasticization, swelling, structural relaxation, post-curing, degradation, hydrolysis, microcracking, and other phenomena typical for polymer composites occur within the polymer matrices of BFRPs [8]. The advantages of BFRPs over fiberglass in terms of strength, fatigue resistance, durability, and the potential for enhancing their mechanical properties through composition modification have also been thoroughly analyzed [8]. In turn, the unique properties of basalt fiber represent a promising solution in materials science and industry for the production of high-quality and durable construction materials and structures, such as BPRP bar, which possesses high strength, corrosion resistance, and light weight. The characteristics of BPRP bar allow its application in industrial, road, and civil construction, as well as in the construction of bridges, various docks, and piers, which are exposed to aggressive environments and corrosion during operation. In global practice, there are numerous studies dedicated to the analysis of the durability of fiberglass bars (GFRB) [9,10,11,12]. It has been established that the tensile strength of GFRP decreases to some extent, and the degree of deterioration is related to aging time, exposure temperature, pH value of the solution, and solar radiation. In the study of Ref. [13], it is shown that three months of exposure to alkali and load can lead to a decrease in the strength of GFRP rods by more than 40%. The resin can provide a protective shell to insulate the alkalis from damaging the fiberglass in the GFRP rods, but destructive substances can still penetrate the rod due to diffusion through the fiber–matrix interface or seepage through cracks or voids in the resin [13].

It is well-known [14] that significant environmental factors affecting polymer materials in open climates include mold fungi, bacteria, and other microbiological agents. Fiber-reinforced polymer (FRP) composite materials are also susceptible to biological degradation. Polymer degradation occurs as microorganisms interact with the material [14]. For example, as shown in Ref. [15], the bacteria *Rhodococcus rhodochrous* and *Ochrobactrum anthropi* increased the surface roughness of the epoxy polymers Araldite LY 5052 and EPON, and caused oxidation of methyl groups in bisphenol A. Biological factors reduce the molecular weight of polymer chains, and the enzymes produced by microbes act as catalysts for oxidation and hydrolytic reactions [16]. The highest microbial activity is observed on the surfaces of the samples [16,17,18]. For instance, biofilm formation on the surface of vinyl ester carbon plastics facilitated bacterial degradation, altering the material’s modulus of elasticity, hardness, onset of thermal degradation, and other properties [17]. Examples of polymer matrix degradation in fiberglass, carbon fiber composites, wood plastics, and hybrid composites under the influence of mold fungi and bacteria, as well as the types of the most active biodeteriorators, are presented in Ref. [18].

Assessing the biological degradation of polymer composite materials in open climatic conditions is a challenging task due to the difficulty of isolating the effect of biodeteriorators on the properties of samples against the backdrop of active influences from temperature, humidity, solar radiation, and other factors. Comparative testing of materials in laboratory or field conditions in the absence of biological factors, as well as by applying active strains to the surface of samples, is considered one of the most promising approaches [18]. When conducting such tests, it is important to identify the types of bacteria and fungi characteristic of the given conditions. Examples of micromycetes found in moderate, moderately warm, and dry subtropical climates are provided by Krivushina [19], and for extremely cold climates by Erofeevskaya [20].

There is particular interest in identifying cryophilic microorganisms capable of colonizing the surface of polymer composite materials in the natural climatic conditions of the Far North. Such a study was conducted by Erofeevskaya [20], where the mechanical properties of untreated and biologically contaminated BFRP bars were compared after exposure in the climate of Yakutsk. The difference in the tensile modulus of elasticity Eb for biologically contaminated BFRP bars with a diameter of 6 mm after 12 months of exposure in Yakutsk was 6%, which falls within the variation coefficient for this parameter and is insufficient for a reliable assessment of the significance of the biological factor.

Therefore, an urgent task is to find other, more sensitive physical indicators capable of detecting the influence of biological factors on the properties of BFRP in the context of combined climatic effects. Such indicators may include moisture diffusion coefficients, determined by gravimetric measurements in steady-state thermal–humidity conditions for the initial and exposed samples [21].

In this regard, the goal of the present work is to investigate the contribution of biological contamination to the moisture transfer properties of BFRP bars exposed in two representative Arctic regions. To achieve this goal, profiled rebar rods with diameters of 6, 8, and 10 mm were used to enable the determination of moisture diffusion coefficients along the reinforcing fibers and in the radial direction.

## 2. Materials and Methods

**Figure 1:**
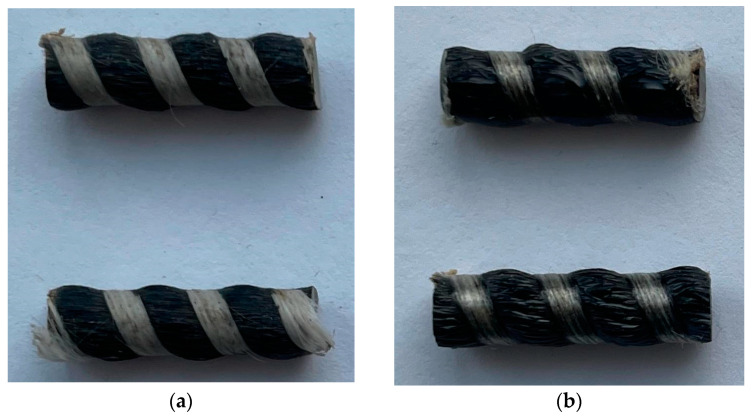
Fragments of BFRP bars with provoked biocontamination, 6 mm in diameter, after 54 months of natural exposure in Yakutsk (**a**) and Tiksi (**b**).

This study used unidirectional basalt-plastic rebars with a periodic profile and diameters of 6 and 8 mm. The rebars were produced from basalt roving RBM 13-2400-4C (TBM LLC, Yakutsk, Russia), supplied by TBM LLC (Yakutsk), in accordance with Technical Specifications 2296-001-86166796-2013, “Non-metallic composite basalt-plastic rebar”. The polymer matrix for the basalt plastic was based on ED-22 epoxy resin, cured at 125 °C with iso-methyl tetrahydrophthalic anhydride (iso-MTHFA) in the presence of the accelerator 2,4,6-tris(dimethylaminomethyl)phenol (UP-606/2). The appearance of the fragments of the investigated BFRP bars with provoked biocontamination, 6 mm in diameter, after 54 months of natural exposure, is shown in Figure 1.

When comparing the samples exposed for 54 months at different climate stations, it was found that the samples exposed in the very cold climate of Yakutsk underwent more significant changes in appearance than those exposed in the Arctic climate of Tiksi. The samples exposed in Yakutsk exhibit more intense lightening, loss of gloss, and breakage of the light wrapping thread (Figure 1a).

Of the various types of samples exhibited for field climate tests in Yakutsk, there were 700 samples of BPA and various plastics in the amount of 61 pieces. Preliminary studies [20] revealed disturbances inhabiting their surface. Liquid biopreparations based on strains of bacteria of the genus *Bacillus* (*B. atropheus*, *Bacillus* sp., and *B. subtilis*) and mold fungi of the genus *Aspergillus* (*A. Niger*), which were dominant in the landscape of the isolated cultures, were manufactured on the basis of the specified microorganisms, which have a neutral effect on each other. Then, 100 BFRP bars were treated with this preparation for 5 days, which was sufficient for biocontamination. For biocontamination, BFRP bar was used (in a ratio of 1:1, with a concentration of at least 1 × 10^9^ cells per 1 cm^3^) in accordance with GOST R IEC 60068-2-10-2009 [22] “Tests for exposure to external factors. Part 2–10. Tests. Test J and guidance: Fungus resistance”. After contamination with spores, the BFRP bars were dried by contact and exposed to the open climatic conditions of Yakutsk and Tiksi, 50 samples at a time, along with untreated bars.

The bar tests were conducted in two representative regions of the Far North—the city of Yakutsk and the settlement of Tiksi. According to climate zoning, the territory of the Republic of Sakha (Yakutia) is classified as a very cold region under GOST 16350-80 [23], with representative locations being the city of Yakutsk, the village of Oymyakon, and the Arctic eastern settlement of Tiksi. The natural and climatic conditions of Yakutia are characterized as extreme in many respects. In terms of the absolute minimum temperature (in the eastern mountain systems—basins, depressions, and other lowlands, temperatures can reach as low as minus 70 °C) and its total duration (ranging from 6.5 to 9 months per year), the republic has no analogues in the Northern Hemisphere.

**Figure 2:**
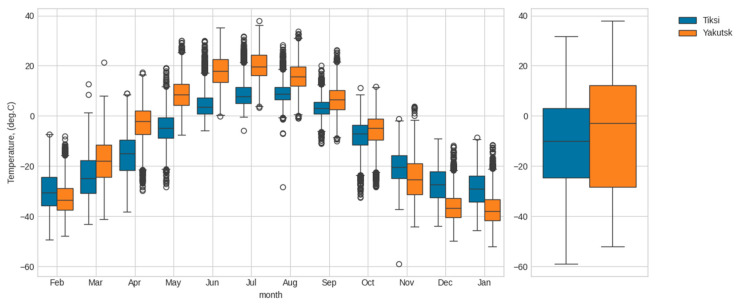
Comparison of the distribution of air temperature by month (on the **left**) and in general (on the **right**) for the period from 2006 to 2024 for Tiksi and Yakutsk.

**Figure 3:**
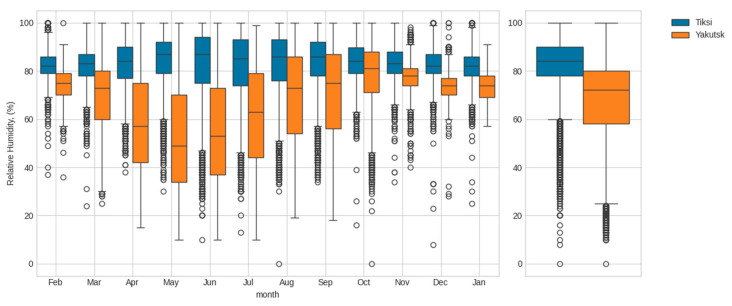
Comparison of the distribution of relative humidity by month (on the **left**) and in general (on the **right**) for the period from 2006 to 2024 for Tiksi and Yakutsk.

Figure 2 and Figure 3 show the average monthly and annual air temperature and relative humidity values as calculated from meteorological data collected over an 18-year period (2006 to 2024).

**Figure 4:**
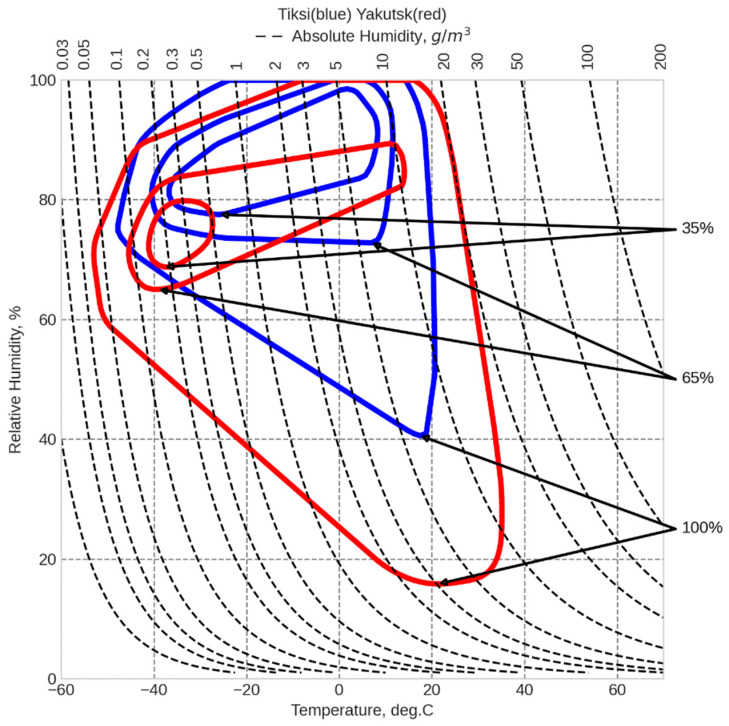
Comparison of the climatogram of the dependence of relative and absolute humidity on temperature for Tiksi and Yakutsk for the period from 2006 to 2024.

Figure 4 shows climatograms of Tiksi and Yakutsk, constructed according to GOST 15150-69 [24] based on meteorological data collected over an 18-year period. The climatogram is a graphical representation of the combinations of air temperature and humidity plotted on a coordinate grid, with values of temperature, relative humidity, and absolute humidity indicated.

Figure 2, Figure 3 and Figure 4 demonstrate significant differences in the thermal and humidity conditions at the material exposure sites. The average annual temperatures of Yakutsk and Tiksi are comparable (−9 °C and −13 °C, respectively). However, the average annual temperature range in Yakutsk is significantly higher than in Tiksi (57 °C vs. 38 °C). Due to the influence of the coastal environment, the annual precipitation (rain and snow) in Tiksi is 320 mm, which is 25% higher than in Yakutsk (240 mm). Moreover, the relative humidity in Tiksi is higher than in Yakutsk throughout the year, with an average annual relative humidity of 83% in Tiksi compared to 68% in Yakutsk. The climatograms in Figure 4 indicate that the thermal and humidity conditions differ the most between Yakutsk and Tiksi during the summer months.

An important climatic characteristic is the annual number of temperature crossings through 0 °C. These crossings cause the melting/freezing of bulk water in the pores, cracks, and defects of polymer composite materials, which leads to increased internal stresses due to volume changes in the frozen water. Such transitions contribute to microcrack formation [25]. According to meteorological data, the average number of these transitions in Yakutsk is twice that in Tiksi (60 vs. 28), which is an important factor to consider when analyzing the aging of BFRP bars.

Tensile strength *σ_b_* and modulus of elasticity *E_b_* measurements were performed on BFRP bars with diameters of 6 mm and 8 mm in both their initial state and after exposure. A Z600 Zwick/Roell universal testing machine was used. Sample loading met the standard requirements [26] and was conducted at room temperature with a loading speed of 5 mm/min. The working length of the sample was 200 mm.

To study moisture transfer, 3 samples of 50, 70, and 100 mm in length were cut from the original and exposed BFRP bars. The cut samples were dried in containers over calcined silica gel for 7 days at 60 °C. After drying, the samples were stored in sealed desiccators above water at a relative humidity of φ = 98 ± 2% for 83 days. The sample mass was measured using analytical scales with an accuracy of 0.1 mg.

## 3. Results

In the study of Ref. [27], the authors provide a detailed examination of the process of bio-damage of BFRP bars by microorganisms. Using microscopy, pores of various sizes, ranging from 21 to 379 µm, as well as cracks ranging from 2 to 6 µm, were observed on the surfaces of the experimental FRPs after biocontamination and climate testing. The presence of such pores and cracks allows microorganisms to freely penetrate the material, fill them, and continue to grow. After 12 months of exposure of the experimental FRP samples at an open site, biological formations in the form of mold hyphae and bacterial cells were found on the material surfaces. The presence of bacterial cells and fungal hyphae may indicate microbial adaptation, the presence of moisture, and other conditions that facilitate their development during exposure in an open ecosystem. Over time, under favorable conditions, microorganisms may also contribute to the degradation of materials and the deterioration of their physical properties. It was established that fungal mycelium was present in the pores formed when the FRP samples were exposed to extremely cold climate conditions. The values of these characteristic temperatures are presented in Table 1.

The lateral bending strength of BFRP bars without provocative biocontamination after 24 months decreases by 5.6%. Biocontamination has a more significant effect, reducing the tensile strength by 15.3% during 24 months of exposure in Yakutsk, and in Tiksi the decrease is even greater—to 16.7%. This indicates that biocontamination has a significantly more destructive effect on the material compared to normal aging without exposure to external factors.

**Figure 5:**
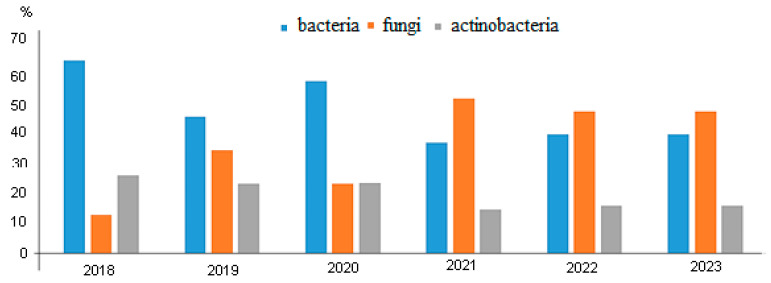
Dynamics of changes in the microbial landscape on the surfaces of FRPs exposed at the climatic testing ground (Yakutsk).

Figure 5 shows the dynamics of changes in the microbial landscape on the surfaces of FRPs exposed at the climatic testing ground (Yakutsk).

Microbiological studies have shown that after 24 months of exposure to extremely low temperatures, bacteria of the genus Bacillus remained immobilized on all 43 samples, while from 10 samples of BFRP bars, strains of mold fungi of the genus Aspergillus were isolated, accounting for 23% of the total viable microflora that remained alive.

A random sample of six BFRP bar samples were selected from both climate zones. The results of determining the effect of biocontamination on the average tensile strength of basalt-plastic reinforcement are presented in Table 2.

As seen in Table 2, after 12, 24, and 54 months of exposure in Yakutsk, the mechanical properties *E_b_*, *σ_b_* of untreated BFRP bars with diameters of 6 and 8 mm changed within the range of 99% to 114% of the initial values, indicating a steady increase. This increase is attributed to the post-curing of the polymer matrix in the BFRP bars under the influence of climatic factors [28]. However, in biologically contaminated rebars, the mechanical properties *E_b_*, *σ_b_* consistently decrease. The extent of this reduction averages 11% and varies from 0% to 21%. Similarly, exposure of biologically contaminated rebars for 54 months in Tiksi results in a decrease in mechanical properties by 2–16% (Table 2).

A possible explanation for this trend is the hypothesis that bacterial cells and fungal mycelium, which penetrate surface irregularities of the profiled rebars under open climate conditions, promote the development of microcracks, and consequently, a degradation of the mechanical properties of the basalt plastic [20].

**Figure 6:**
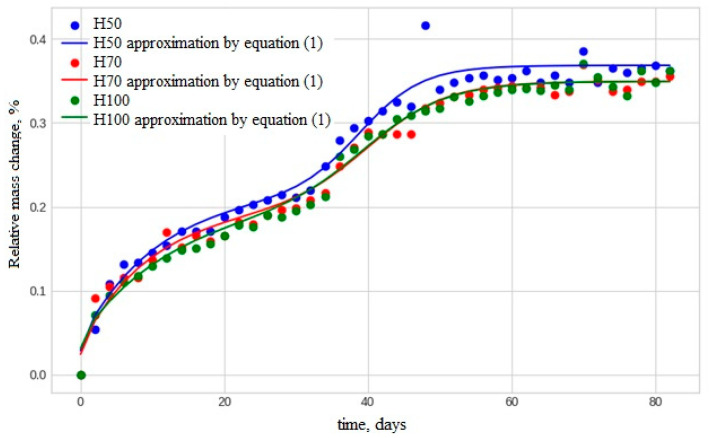
Moisture sorption kinetics in initial BFRP bar samples with a diameter of 6 mm and lengths of H = 50, 70, and 100 mm.

A comparison of the BFRP bar conditions after exposure in two different climatic regions was conducted using thermogravimetric analysis. It was found that the moisture absorption kinetics in the tested rebars are characterized by two distinct stages of moisture saturation. A characteristic example is shown in Figure 6, which illustrates the moisture sorption kinetics in initial basalt-plastic rebar samples with a diameter of 6 mm and lengths of 50, 70, and 100 mm when held in humid air at 60 °C. During the first 30 days, typical Langmuir sorption is observed [29]. After this, a second stage of increase in w(t), occurs, which is generally indicative of a chemical reaction [30]. A key feature of the chemical reaction is its threshold behavior, as a certain concentration of accumulated water is necessary to initiate the reaction.

Therefore, the amount of sorbed moisture in the sample *w(t)* is the sum of two components:

(1) $$w\left(t\right)=w_{L}\left(t\right)+w_{C}\left(t\right),$$

where *w_L_*(*t*) is the amount of moisture accumulated according to the classical Langmuir diffusion law, and *w_C_*(*t*) is the amount of moisture absorbed by the sample during the chemical reaction.

**Figure 7:**
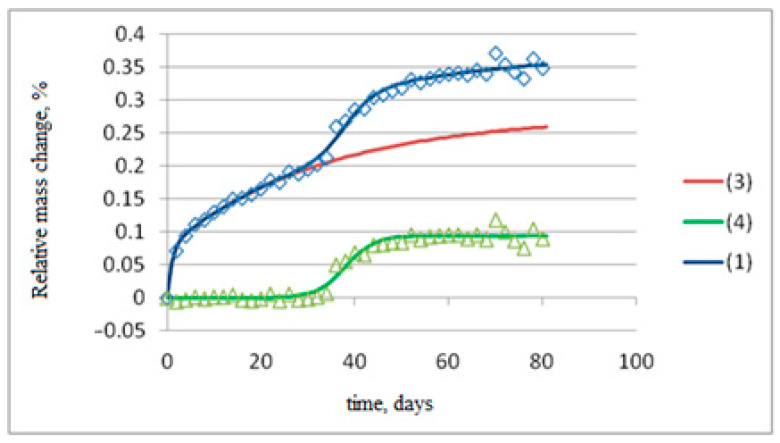
Moisture sorption modeling of basalt-plastic rebar samples with a length of 100 mm and a diameter of 6 mm using the following equations: (1)—blue curve, (3)—red curve, (4)—green curve.

Moisture sorption modeling is illustrated in Figure 7 for samples with a length of 100 mm and a diameter of 6 mm (experimental values of *w* for three parallel samples are marked with symbols).

In the first stage (red line in Figure 7), moisture diffusion into the anisotropic rods is modeled by a parabolic partial differential equation for a finite cylinder with radius *R* and length *H =* 2*h* [31,32].

(2) $$\frac{\partial w\left(r,z,t\right)}{\partial t}=D_{r}\left(\frac{\partial^{2}w\left(r,z,t\right)}{{\partial r}^{2}}+\frac{1}{r}\frac{\partial w\left(r,z,t\right)}{\partial r}\right)+D_{z}\frac{\partial^{2}w\left(r,z,t\right)}{{\partial z}^{2}}\;0\leq r\leq R,-h\leq z\leq h,\;0\leq t\;w\left(r,z,0\right)=0,\;w\left(R,z,t\right)=w\left(r,-h,t\right)=w\left(r,h,t\right)\;w\left(0,z,t\right)<\infty$$

The solution to Equation (2) is as follows:

(3) $$w_{L}\left(t<t_{0}\right)=w_{0}\left(1-Gexp\left[-\beta t\right]-B\sum \left[t,D_{r},D_{z}\right]\right),\;\sum \left[t,D_{r},D_{z}\right]=\sum_{n=1}^{\infty }\sum_{m=1}^{\infty }L_{n}L_{m}exp\left[-\left(\mu_{n}^{2}\frac{D_{r}}{R^{2}}+\mu_{m}^{2}\frac{4D_{z}}{H^{2}}\right)t\right],\;L_{n}=\frac{4}{\mu_{n}^{2}},\;L_{m}=\frac{2}{\mu_{m}^{2}},\;J_{0}\left(\mu_{n}\right)=0,\;\mu_{m}=\left(2m-1\right)\frac{\pi }{2},\;G=\frac{\gamma }{\gamma +\beta },\;B=\frac{\beta }{\gamma +\beta }.$$

Here:

*w*_0_—the maximum moisture content, %;

*D_z_*—diffusion coefficient along the length, mm^2^/day;

*D_r_*—diffusion coefficient along the radius, mm^2^/day;

*G*, *B*—probabilities;

$J_{0}\left(\mu_{n}\right)$—Bessel function of the first kind and zero order;

*t*—duration of moisture saturation;

β—probability per unit time of conversion from bound water to mobile water;

γ—probability per unit time of conversion from mobile water to bound water.

In the second stage (green line in Figure 6), the contribution of the chemical reaction to the amount of sorbed moisture is described by an equation in the form of a smoothed step function [21].

(4) $$w_{C}\left(t\right)=B_{c}/\left(1+exp\left(-k_{c}\left(t-t_{c}\right)\right)\right)$$

where:

*B_C_*—the contribution of the chemical reaction to the overall mass increase;

*k_C_*—a parameter correlated with the chemical reaction rate constant;

*t*_0_—the time of maximum mass change due to the chemical reaction.

By summing the terms calculated using Equations (3) and (4), we obtain the final dependence *w*(*t*), represented by the blue line in Figure 7. Mathematical processing demonstrated that, for the entire series of basalt-plastic rebar samples with diameters of 6 mm, 8 mm, and 10 mm, and lengths of 50 mm, 70 mm, and 100 mm, exposed in Tiksi and Yakutsk, the approximation of the experimentally obtained kinetic curves *w*(*t*) with models (3) and (4) was successful, with determination coefficients *R^2^* ranging from 0.89 to 0.96.

**Figure 8:**
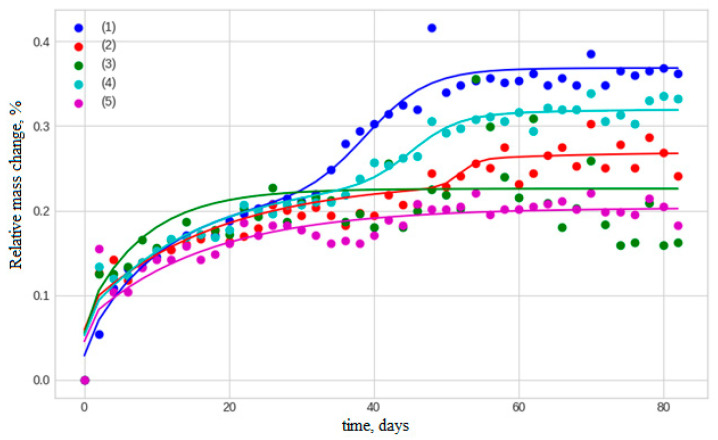
Influence of exposure duration on the moisture sorption kinetics of untreated and biologically contaminated basalt-plastic rebar samples with a diameter of 6 mm and a length of H = 50 mm, where: (1) initial state, (2) after 24 months in Tiksi (untreated), (3) after 54 months in Tiksi (untreated), (4) after 24 months in Tiksi (with biological contamination), (5) after 24 months in Yakutsk (with biological contamination).

Selected examples of the influence of exposure duration on the moisture sorption kinetics of untreated and biologically contaminated basalt-plastic rebars are shown in Figure 8. After 83 days of gravimetric measurements, the moisture content *w* of samples with a diameter of 6 mm and a length of 50 mm reached 0.37%, and then decreased to 0.26% and 0.22% after 24 and 54 months of exposure in Tiksi, respectively. In biologically contaminated samples exposed for 24 months in Tiksi, the moisture content was 0.32%.

The summarized results of moisture transfer modeling for the entire set of tested basalt-plastic rebar samples are presented in Table 3.

After 24–54 months of exposure, the maximum moisture content *w*_0_ in BFRP bars decreased from 0.22% to 0.16%. In biologically contaminated samples, *w*_0_ decreased further to 0.15%. The reduction of parameter *B* from 0.32 to 0.18 suggests that the conversion of bound water to mobile water in exposed samples becomes less probable, likely due to a decrease in the number of hydrophilic sites as a result of post-curing of the epoxy matrix.

The data in Table 3 confirm the general trend discussed in Ref. [25], where the moisture diffusion coefficient along the reinforcement direction, *D_z_*, is almost two orders of magnitude (79 times) higher than the equivalent coefficient in the radial direction, *D_R_*. In Ref. [8], it was shown that, under open climatic conditions, daily and seasonal thermal cycles and fluctuations in relative humidity induce physicochemical transformations in BFRPs, leading to degradation and the accumulation of micro-damage in the surface layers. Consequently, after 54 months of climate-induced aging in Tiksi, the diffusion coefficients *D_z_* and *D_R_* increased by 1.6 and 2.3 times, respectively. This effect may be associated with an increase in free volume within the epoxy matrix of the BFRP bar.

The role of biological contamination in changing moisture transfer properties is also noteworthy. After 24 months of exposure of untreated BFRP bar in Tiksi, *D_R_* reached 0.023 mm^2^/day (an increase of 1.6 times). The presence of bacteria and mold fungi enhances the effect of climatic factors, resulting in *D_R_* = 0.041 mm^2^/day over the same exposure period—an increase of 2.9 times. In Yakutsk, 24 months of aging raised this increase to 3.4 times (Table 3).

It is also important to note the reduction in the contribution of the chemical reaction to the overall maximum mass increase of the exposed samples, *w*_0_. This conclusion is supported by the decrease in the *B_c_* parameter from 0.19% to 0.06% (Table 3). Meanwhile, mold fungi and bacteria do not affect the time of maximum mass change due to the chemical reaction, *t*_0_.

**Figure 9:**
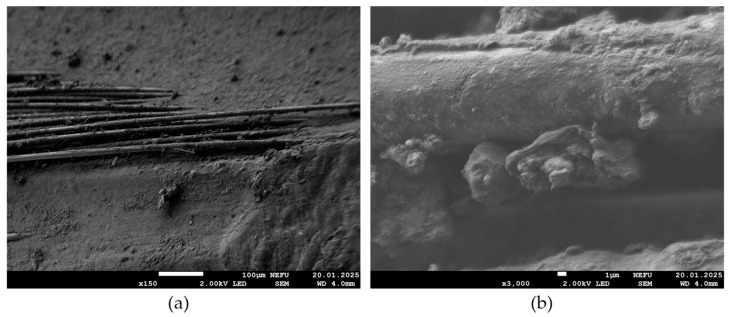
Microstructure of BFRP bar with provocative biocontamination after exposure in Yakutsk for 54 months, magnifications: (**a**) 150×, (**b**) 3000×.

Figure 9 shows a microstructure of a fragment of BFRP bar after provocative biocontamination and exposure for 54 months in the open ecosystem of Yakutsk. The micrograph (b) shows a spore of mold fungi of the genus *Aspergillus* (1), which demonstrates characteristic structural features indicating the impact of the environment and, possibly, stress caused by interaction with other microorganisms. Additionally, the image shows two bacterial cells (1); one of them has a rod-shaped form, and the other is coccus-shaped, which indicates the diversity of microbial flora in the sample under study. In addition, the figure shows a fragment of biofilm (2), which is an aggregation of microbes that unite on the surface and form a protective structure. Overall, the micrograph in Figure 9 not only captures the current state of the sample after long-term biocontamination, but also provides valuable information about the interactions of various microorganisms in the BFRP bar, highlighting the importance of studying microbial activity to understand the degradation processes and dynamics of biological objects.

After collecting additional experimental data, we plan to conduct an extended analysis of the influence of exposure conditions (temperature, relative humidity, microorganism composition, etc.) on the retention of mechanical properties and changes in moisture diffusion coefficients in anisotropic BFRP bars along the reinforcement direction and in the radial direction.

## 4. Conclusions

After 12, 24, and 54 months of exposure in the sharply continental climate of Yakutsk and the humid Arctic climate of Tiksi, the tensile strength and modulus of elasticity of untreated BFRP bar samples increased by 5–14% due to post-curing of the epoxy matrix. In biologically contaminated samples, these indicators decreased by an average of 11%.

The moisture diffusion coefficients along the reinforcement direction and in the radial direction of the basalt-plastic rebar were found to be sensitive to the impact of microorganisms under the variable temperature and humidity conditions of the Arctic climate.

In the presence of mold fungi and bacteria, there was a significant increase in the moisture diffusion coefficient in the radial direction.

The results of the moisture transfer kinetics studies presented in this work are of clear scientific and practical interest and call for further, more extensive research.

## Figures and Tables

**Table 1:** Effect of biocontamination on the transverse bending strength of 6 mm basalt-plastic reinforcement bar exposed in Yakutsk and Tiksi.

Exposure Duration, Months	Material Condition	*σ_f_*, MPa **
0	Initial state	1041 ± 9.4/100
24	Untreated	983 ± 8.8/94
24	Biologically contaminated	882 ± 7.9/85
24 *	Biologically contaminated	877 ± 7.9/84

* Material exposed in Tiksi. ** Numerator—absolute value; denominator—retention coefficient *k_R_* = *R_t_/R*_0_∙100, %, where *R_t_* represents the values of *E_b_*, *σ_b_* after the exposure time *t*, *R*_0_ represents the initial values of *E_b_*, and *σ_b_* before exposure.

**Table 2:** Effect of biocontamination on the tensile strength of basalt-plastic rebars exposed in Yakutsk and Tiksi.

Exposure Duration, Months	Material Condition	*E_b_*, GPa **		*σ_b_*, MPa **	
		6 mm	8 mm	6 mm	8 mm
0	Initial state	53.2 ± 0.5/100	50.8 ± 0.5/100	1120 ± 11.2/100	1000 ± 10/100
12	Untreated	52.7 ± 5.3/99	51.2 ± /101	1210 ± 12.1/108	1020 ± /102
12	Biologically contaminated	49.7 ± 4.9/93	44.7 ± /88	910 ± 9.1/82	792 ± 7.9/79
24	Untreated	52.7 ± 5.3/99	51.2 ± 5.2/101	1200 ± 11.8/107	1080 ± 10.8/108
24	Biologically contaminated	48.8 ± 5.1/92	45.1 ± 4.4/89	920 ± 9.2/82	931 ± 9.3/93
54	Untreated	53.5 ± 5.3/101	52.1 ± 5.2/103	1280 ± 12.8/114	1080 ± 10.8/108
54	Biologically contaminated	53.0 ± 5.3/100	46.7 ± 4.5/92	990 ± 9.9/88	870 ± 8.7/87
54 *	Biologically contaminated	47.2 ± 4.8/89	50.0 ± 4.9/98	942 ± 9.4/84	853 ± 8.5/85

* Material exposed in Tiksi. ** Numerator—absolute value; denominator—retention coefficient *k_R_* = *R_t_/R*_0_*∙*100, %, where *R_t_* represents the values of *E_b_*, *σ_b_* after the exposure time *t*, *R*_0_ represents the initial values of *E_b_*, and *σ_b_* before exposure.

**Table 3:** Average size-specific parameters of models (3) and (4).

Test Location	*t*, Months	Bar Condition	*w*_0_, %	*D_z_* mm^2^/Day	*D_R_* mm^2^/Day	*B*	*G*	*B_c_*, %	*K_c_*, 1/Day	*t_c_*, Day
Initial	0	Untreated	0.22	1.1	0.014	0.079	0.32	0.19	0.16	36
Tiksi	24	Untreated	0.16	1.4	0.023	0.12	0.26	0.10	0.10	46
Tiksi	54	Untreated	0.16	1.8	0.032	0.099	0.24	0.10	0.14	43
Tiksi	24	Bio *	0.15	1.2	0.041	0.079	0.20	0.07	0.10	43
Yakutsk	24	Bio *	0.15	1.2	0.047	0.10	0.18	0.06	0.18	43

* Biologically contaminated with a mix of bacteria and fungi before exposure.

## Data Availability

The original contributions presented in this study are included in the article; further inquiries can be directed to the corresponding author.
